# Ionogels Based on Poly(methyl methacrylate) and Metal-Containing Ionic Liquids: Correlation between Structure and Mechanical and Electrical Properties

**DOI:** 10.3390/ijms17030391

**Published:** 2016-03-16

**Authors:** Kerstin Zehbe, Matthias Kollosche, Sebastian Lardong, Alexandra Kelling, Uwe Schilde, Andreas Taubert

**Affiliations:** 1 Institute of Chemistry, University Potsdam, Karl-Liebknecht-Straße 24-25, 14476 Potsdam-Golm, Germany; kerstin.zehbe@uni-potsdam.de (K.Z.); sbake@uni-potsdam.de (S.L.); kelling@chem.uni-potsdam.de (A.K.); us@chem.uni-potsdam.de (U.S.); 2 Institute of Physics & Astronomy, University Potsdam, Karl-Liebknecht-Straße 24-25, 14476 Potsdam-Golm, Germany; kollosch@uni-potsdam.de

**Keywords:** ionic liquids, ionogels, ionic conductivity, mechanical properties, microstructure, phase separation

## Abstract

Ionogels (IGs) based on poly(methyl methacrylate) (PMMA) and the metal-containing ionic liquids (ILs) bis-1-butyl-3-methlimidazolium tetrachloridocuprate(II), tetrachloride cobaltate(II), and tetrachlorido manganate(II) have been synthesized and their mechanical and electrical properties have been correlated with their microstructure. Unlike many previous examples, the current IGs show a decreasing stability in stress-strain experiments on increasing IL fractions. The conductivities of the current IGs are lower than those observed in similar examples in the literature. Both effects are caused by a two-phase structure with micrometer-sized IL-rich domains homogeneously dispersed an IL-deficient continuous PMMA phase. This study demonstrates that the IL-polymer miscibility and the morphology of the IGs are key parameters to control the (macroscopic) properties of IGs.

## 1. Introduction

Ionic liquids (ILs) have found application in many different fields ranging from chemical synthesis to sensing and membranes [1,2,3]. Often the IL cation is used to infer supramolecular order to the IL, such as in ionic liquid crystals (ILCs) [4,5]. In contrast, the anion often significantly contributes to the physico-chemical properties and reactivity such as catalytic activity, magnetism, luminescence, or electrochemical response [6,7,8,9]. Consequently, the proper choice of IL anions and cations provides access to a wide variety of (multi)functional ILs. Moreover, ILs with more than one anion or cation, that is, mixtures of ILs, can further enhance these properties [5,10,11,12,13,14,15,16,17,18].

ILs combine high ionic conductivity, broad electrochemical stability windows, and good thermal stability [19,20,21] and are thus, among others, interesting for electrochemical applications such as batteries and fuel cells [22,23,24,25]. Moreover, ILs containing (transition) metals often show an interesting and useful redox behavior that can be exploited for sensing [26,27] or catalysis [28,29,30,31]. Arguably, the most important metal-based IL components are the tetrahalidometallate [MX_4_]^*n*−^ (M = metal, X = Cl, Br, I) anions. These anions allow the adjustment of chemical and physical properties by proper choice of metal and halide ion or by refining their interactions and structure via hydrogen bonds or via aromatic building blocks [32,33]. The resulting ILs are therefore interesting for a wide variety of applications [19]. For example, several tetrahalidometalate ILs have shown high catalytic activities. Gärtner and coworkers showed that tetrachloridoferrate(III) ILs are useful reagents in organic synthesis [34,35]. Other examples support these observations [36,37].

One of the issues in these systems is that the IL is not easily separated from the reaction mixture and IL recycling may become a challenge. One approach to remove a catalytically active IL from a mixture is thermally induced phase separation [38,39,40]. A further alternative for catalyst management is the incorporation of ILs in a matrix, for example yielding advanced hybrid materials called ionogels or ion-gels (IGs) [41,42,43,44]. Because IGs are highly functional and adaptable materials combining the properties of the IL and the matrix, they are attractive for use in catalysis, but also in batteries, fuel cells, sensors, *etc.* For example Xie *et al.* [38] reported that the magnetic IL 1-butyl-3-methyl imidazolium tetrachloridoferrate(III), [Bmim][FeCl_4_], has been successfully incorporated into a poly(methyl methacrylate) (PMMA) matrix, yielding a weakly paramagnetic IG. Bagdahn and Taubert showed that PMMA-based IGs containing 1-butyl-3-methyl imidazolium tetrachloridocuprate(II), [Bmim]_2_[CuCl_4_], can successfully be electrospun, thus providing access to high-surface-area IGs with additional functionalities [42,45,46,47]. Moreover, IGs often exhibit higher stabilities than the pure components [29,42,48,49,50,51,52,53]. Overall, these data show that, in principle, the combination of ILs and a suitable matrix should offer virtually unlimited potential for materials design. Surprisingly, many authors claim that IGs have a high application potential, yet there are very little data on mechanical [54] and electrical properties [45,49,55,56,57]. This is particularly pronounced for IGs based on metal-containing ILs, which is intriguing because (metal-based) IGs may be attractive as a high energy density polymer for soft electronics, especially in transistors, capacitors, and varistors [58,59,60] or for hard-soft integration materials [61,62]. The current study therefore focuses on a set of model IGs composed of poly(methyl methacrylate) (PMMA) and the ILs bis-1-butyl-3-methlimidazolium tetrachloride cuprate(II), [Bmim]_2_[CuCl_4_], tetrachloride cobaltate(II), [Bmim]_2_[CoCl_4_], and tetrachloride manganate(II), [Bmim]_2_[MnCl_4_], to evaluate the effects of IL content and chemistry on mechanical and dielectric properties and to determine if the structure and morphology of the IG can be correlated to the macroscopic properties elongation at break and conductivity.

## 2. Results

### 2.1. Ionic Liquids

The ILs [Bmim]_2_[CuCl_4_], [Bmim]_2_[CoCl_4_], and [Bmim]_2_[MnCl_4_] were synthesized according to published protocols [63,64,65]. Single crystals were obtained by diffusion of methyl-tert-butyl ether (MTBE) into ethanolic solutions of the respective IL [66]. The crystallographic data are summarized in Table 1 and confirm results from the literature [67].

**Figure 1:**
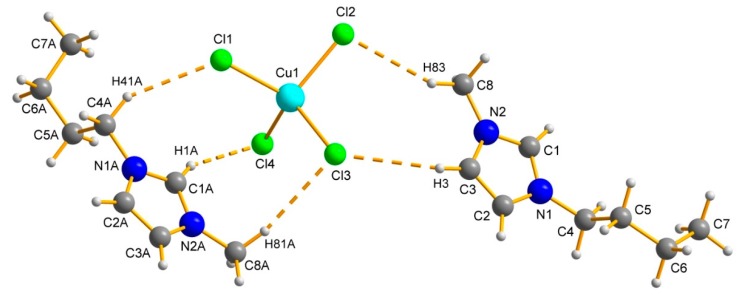
The asymmetric unit of [Bmim]_2_[CuCl_4_] with numbering scheme and H–Cl interactions.

[Bmim]_2_[CuCl_4_], [Bmim]_2_[CoCl_4_] and [Bmim]_2_[MnCl_4_] are isostructural. All ILs have a monoclinic crystal system and similar unit cell parameters. As the covalent atom radius of manganese is the largest of the series, the dimension of the unit cell is the largest, too. In all cases, the asymmetric unit contains two independent cations and a slightly distorted tetrahedral [CuCl_4_]^2−^, [CoCl_4_]^2−^ or [MnCl_4_]^2−^ anion, respectively. As a representative example the structure of [Bmim]_2_[CuCl_4_] is shown in Figure 1.

The largest distortion from the perfect tetrahedron is observed in [CuCl_4_]^2−^ with Cl–Cu–Cl bond angles ranging from 100.98(5) to 126.61(5)°. The Cl–M–Cl bond angles ranges from 107.23(4) to 11.60(4)° in [BMIM]_2_[CoCl_4_] and from 106.61(3) to 11.93(2)° in [Bmim]_2_[MnCl_4_], respectively. The average M–Cl bond lengths are 2.249 Å (Cu), 2.276 Å (Co) and 2.362 Å (Mn). For bond lengths and angles, see Tables S1 to S3 (Supplementary Materials). These bond lengths and angles are very similar to those found in the Cambridge Structural Database for other Cu(II), Co(II) and Mn(II) salts containing tetrahedral [CuCl_4_]^2−^, [CoCl_4_]^2−^, and [MnCl_4_]^2−^ anions. Moreover, all compounds exhibit a large number of non-classical C–H···Cl hydrogen bonds. Tables S4–S6 contain the details of the hydrogen bond geometry. In addition, π···π, C–H···π and M–Cl··· π interactions (Tables S7–S15) contribute to the structure and influence melting points and other properties. Table 1 summarizes all crystallographic and refinement data for the ILs investigated in this work.

**Figure 2:**
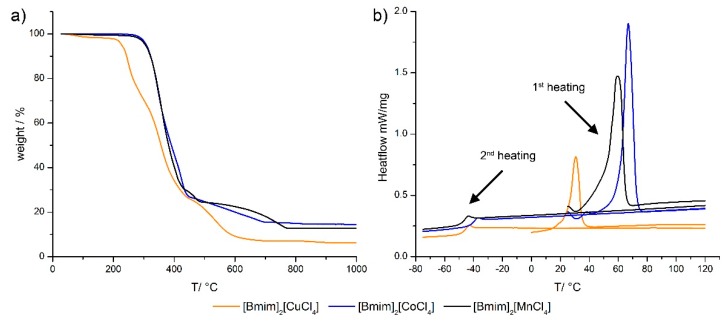
Thermogravimetric analysis (TGA) data (**a**) and differential scanning calorimetry (DSC) heating curves (**b**) of the three ILs.

The thermal behavior of [Bmim]_2_[CuCl_4_], [Bmim]_2_[CoCl_4_] and [Bmim]_2_[MnCl_4_] (Table 2) was investigated by thermogravimetric analysis (TGA) and differential scanning calorimetry (DSC) (Figure 2). The TGA data of [Bmim]_2_[CoCl_4_] and [Bmim]_2_[MnCl_4_] exhibit no significant existence of water or other solvent as only a negligible mass loss of 0.2%–1.2% is observed until 150 °C. [Bmim]_2_[CuCl_4_] shows a mass loss of 1.2% until 150 °C suggesting the presence of some residual solvent in this particular IL.

Presumably, the organic ring system in [Bmim]_2_[CuCl_4_] starts to decompose at *ca.* 210 °C which is indicated by a mass loss of 2.4% From this point to 600 °C the organic moieties undergo full degradation and the residual mass remains constant at approximately 9%. This is much smaller than the theoretical value of 20% for the final product CuCl, which would be expected to form under the chosen conditions. For [Bmim]_2_[CoCl_4_] and [Bmim]_2_[MnCl_4_] the decomposition of the organic cation occurs from 276 to 750 °C with a mass loss of around 74%. Up to 1000 °C, no further mass losses can be detected in these two cases and the residual mass of *ca.* 14% is consistent with the formation of CoCl_2_ and MnCl_2_.

DSC shows that all ILs have a similar phase behavior. During the first heating cycle, a clear melting peak is visible at approximately 26, 48 and 49 °C. During the second heating cycle only a glass transition (*T*_g_) can be observed; it is in the range of −42 to −50 °C for all ILs. All subsequent heating and cooling cycles show a *T*_g_ signal between −40 and −50 °C. Table 2 summarizes the thermal properties of [Bmim]_2_[CuCl_4_], [Bmim]_2_[CoCl_4_] and [Bmim]_2_[MnCl_4_].

The magnetic susceptibility data at room temperature correspond to an effective magnetic moment (µeff) of the copper ion of 1.78 BM and the manganese ion of 5.42 BM according to the literature value [68]. It is also known that the tetrahalido ion, [CoCl_4_]^2−^ shows a magnetic moment of 4.56 BM which assumes the value of 4.47 BM for [Bmim]_2_[CoCl_4_] [69]. These results show that there is no strong evidence of a metal-metal interaction, similar to data by Strauch *et al.* [70] who observed very weak metal-metal interactions in similar compounds. Table 2 summarizes the thermal properties and magnetic moments of [Bmim]_2_[CuCl_4_], [Bmim]_2_[CoCl_4_] and [Bmim]_2_[MnCl_4_].

### 2.2. Ionogels (IGs)

**Figure 3:**
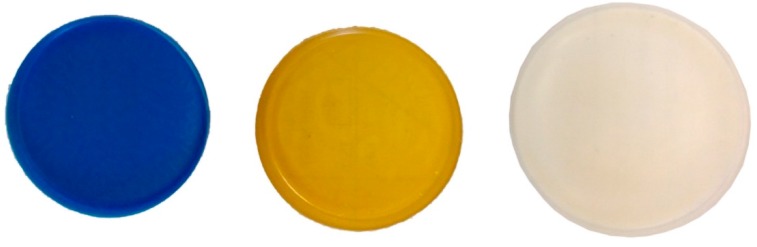
Photograph of ionogels (IGs) used in this study. IGs are based on [Bmim]_2_[CoCl_4_] (**left**), [Bmim]_2_[CuCl_4_] (**middle**), [Bmim]_2_[MnCl_4_] (**right**). The ionic liquid (IL) loading is 20%.

The metal-containing IGs were synthesized via solution casting of IL and PMMA from acetonitrile. All films have a thickness of 1–2 mm and are flexible, transparent, and colored according to the metal-based IL contained within the IG. The IGs contain a maximum of 40% of the IL. Above 50% of IL the composites become very soft and viscous. Figure 3 shows photographs of the IGs. IGs will be labeled as follows: ^M^IG^XX^ where M = Mn, Co, Cu indicates the type of IL in the IG and XX = 10, 20, 30 or 40 indicate the fraction of IL in the IG.

**Figure 4:**
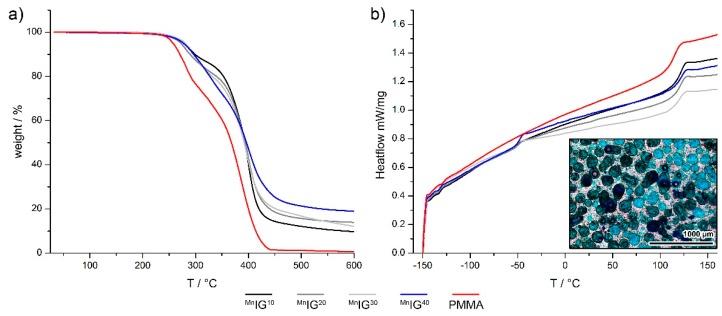
(**a**) TGA curves of poly(methyl methacrylate) (PMMA) and ^Mn^IG^10^ to ^Mn^IG^40^ obtained from measurements under nitrogen and at a heating rate of 10 K/min. TGA data of the other IGs are shown in Figure S1 (Supplementary Materials); (**b**) DSC curves of IGs (^Mn^IG^10−40^) and PMMA. Inset in (**b**) shows an optical micrograph of ^Co^IG^20^ with the blue domains being the IL and the transparent domains being the PMMA.

Figure 4a shows representative TGA data of the IGs with different fractions of [Bmim]_2_[MnCl_4_] and the pure PMMA. Pure PMMA decomposes in a two-step process. The first weight loss of 30.3% is observed between 235 and 320 °C. We assign this weight loss to PMMA chain fragmentation. Since 235 °C is somewhat higher than the ceiling temperatures of PMMA found in the literature [71], it may be possible that the first fragments are not volatile but only the decomposition products formed above *ca.* 235 °C are small enough to become volatile. A second decomposition step can then be observed between 320 and 450 °C, ultimately leading to the complete decomposition of the PMMA.

The IGs decompose with an analogous two-step process with a higher residual mass than in the pure PMMA. The temperature windows of decomposition are, however, roughly comparable to those just described for PMMA.

^Mn^IG^40^ shows a total mass loss of 76%, which is relatively close to the theoretical value of 72% in the case of only MnCl_2_ formation. The difference may be due to the partial formation of oxides. The residual mass consistently decreases in 2% steps with decreasing IL fraction from ^Mn^IG^40^ to ^Mn^IG^10^. TGA data of ^Co^IG^40^ shows a mass loss of 79%, which in the vicinity of the theoretical value of 71% for the formation of CoCl_2_. Again, decreasing IL fractions lead to decreasing residual mass at 1000 °C. The same observation can be made for CuIG40, which shows a weight loss of 74% and a corresponding decrease in residual mass with decreasing IL content. Overall, TGA confirms that the IGs do not contain significant amounts of water or organic solvent and that addition of the IL stabilizes the IGs to some extent; this is consistent with the literature [72].

Figure 4b shows the differential scanning calorimetry (DSC) traces (second heating) of select IGs (^Mn^IG^10−40^). The DSC thermogram of the same IGs shows two endothermic steps at −50.2 and at 114.3 °C, indicating two independent glass transitions of the IL and the PMMA. Indeed, the presence of two separate phases is confirmed by optical microscopy.

DSC shows that the IGs exhibit two glass transitions. The first one is at around −40 to −50 °C and can be assigned to the ILs. The second glass transition is at 114–116 °C and can be assigned to the PMMA matrix. Table 3 summarizes the results from DSC.

**Figure 5:**
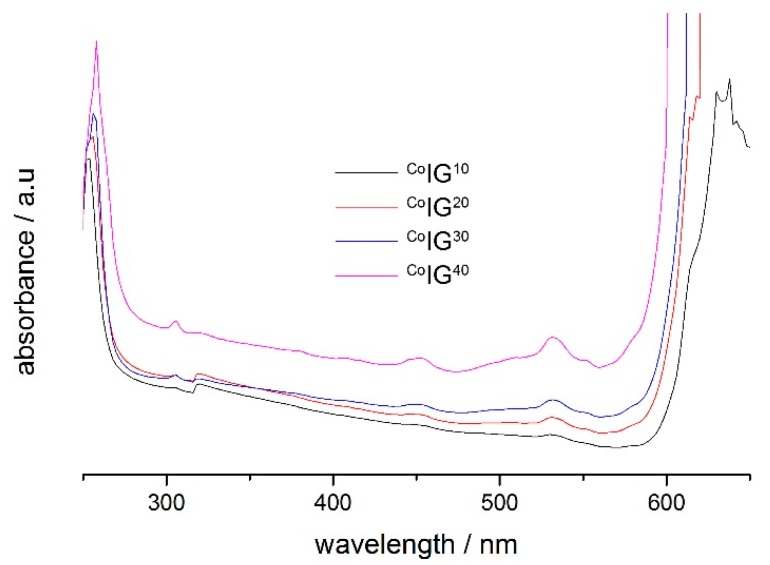
Solid-state-UV-spectra of ^Co^IG^10−40^.

Figure 5 shows the corresponding solid-state UV/Vis data obtained from reflection measurements. Pure PMMA is transparent and shows no absorption between 300 and 900 nm. UV-spectra of ^Co^IG^XX^ shows three absorption bands at 453, 530 and 553 nm originating from the [CoCl_4_]^2−^ anion [73]. The optical properties of the [CoCl_4_]^2−^ are essentially caused by allowed d-d transitions. The electronic ground state of Co^2+^ (3d^7^) is ^4^F and the first excited state is ^4^P. The three main bands observed at room temperature for the Co^2+^ spectra are the splitting of the ground state ^4^F to the three terms ^4^A_2_, ^4^T_2_ and ^4^T_1_.

All ^Cu^IG^10−40^ samples show very strong absorption and no spectra could be observed. For the ^Mn^IG^10−40^ materials no absorption could be observed; this is likely due to the fact that in Mn(II), all electronic transitions are forbidden [74].

**Figure 6:**
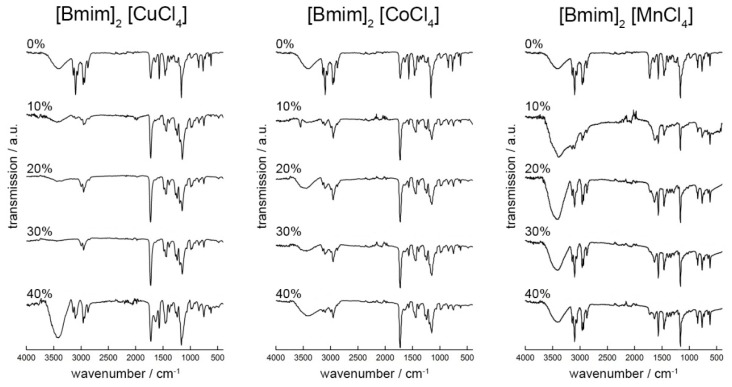
IR spectra of IGs with different fractions of [Bmim]_2_[CuCl_4_] (^Cu^IG^1^^0−4^^0^), [Bmim]_2_[CoC l_4_] (^C^^o^IG^1^^0−4^^0^) and [Bmim]_2_[MnCl_4_] (^Mn^IG^1^^0−4^^0^). Spectra are shifted vertically for better visibility.

Figure 6 shows representative attenuated total reflection infrared (ATR-IR) spectra of pure PMMA and ^Cu^IG^XX^, ^C^^o^IG^XX^, and ^Mn^IG^XX^. IR spectra of pure PMMA show intense bands at 1153 cm^−1^ from the C–O stretching vibration, at 1725 cm^−1^ from the C=O stretching vibration, at 2947 cm^−1^ from the symmetric C–H stretching vibration of the methyl ester, and a band at 3092 cm^−1^ from the aliphatic C–H stretching vibration.

IR spectra of pure [Bmim]_2_[CuCl_4_] show bands at 3140 and 3092 cm^−1^ that are assigned to aromatic C–H stretching vibrations [75] and bands at 2947 and 2858 cm^−1^ are assigned to aliphatic C–H stretching vibrations. Strong bands at 1556 and 1164 cm^−1^ are due to imidazolium ring stretching vibrations. Less intense bands at 830 and 738 cm^−1^ are from C–H in plane bending vibrations. The band at 614 cm^−1^ is likely due to bending vibrations of the aromatic ring. The spectra of the corresponding Co- and Mn-based ILs and IGs are very similar. The only exception is the C=O stretching vibration at 1624 cm^−1^ in the [Bmim]_2_[MnCl_4_]. This small shift likely indicates that there are different interactions between the cation and the metal containing anion and the matrix depending on the metal cation: while [Bmim]_2_[CuCl_4_] and [Bmim]_2_[CoCl_4_] appear to be indifferent to volume fraction changes, [Bmim]_2_[MnCl_4_] seems somewhat more sensitive to the surroundings. Possibly this is due to a slightly better miscibility with PMMA.

**Figure 7:**
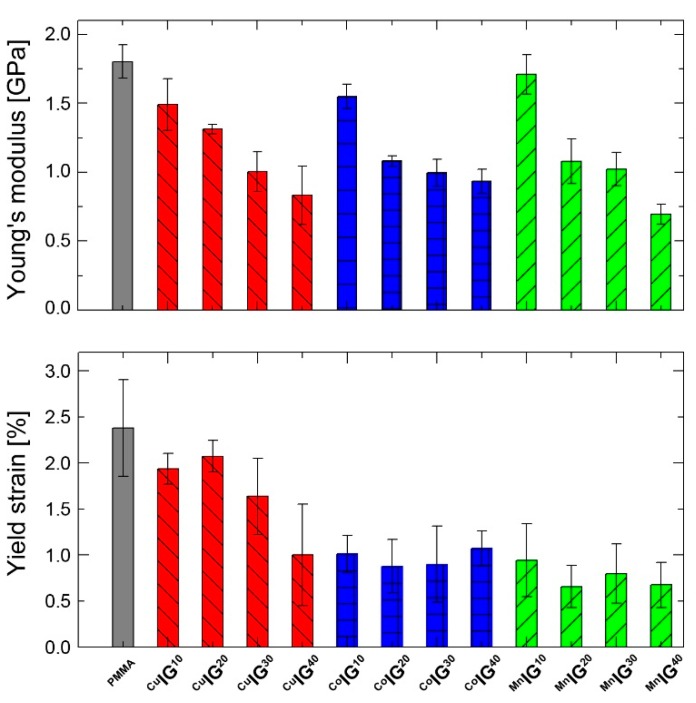
(**Top**) Young’s moduli of PMMA and ^CuM^IG^10−40^, ^Co^IG^10−40^ and ^Mn^IG^10−40^; (**Bottom**) Yield strains of all samples.

Figure 7 summarizes the elastic moduli (Young’s moduli) determined from stress-strain curves of the different IGs. The Young’s modulus of the freshly prepared unfilled PMMA is 1.800 GPa. It decreases with increasing amounts of [Bmim]_2_[CuCl_4_] to 1.490, 1.312, 1.002, and finally 0.832 GPa at 40% of [Bmim]_2_[CuCl_4_]. This corresponds to a reduction of *ca.* 50% compared to the unfilled PMMA. The addition of either the Co- or Mn-containing IL results in a significant reduction of Young´s modulus. The most significant reduction is observed between 10% and 20% of IL loading. The reduction of Young´s modulus at 40% of IL is 62% ([Bmim]_2_[MnCl_4_]) and 49% ([Bmim]_2_[CoCl_4_]), respectively, compared to pure PMMA.

Figure 7b shows yield strain data for the same samples. Consistent with the change of Young´s modulus, the PMMA ruptures at 2.4% while all IGs fail at lower strains. In contrast to the Cu-based systems, where low IL fractions do not lead to a strong reduction of the yield strain, already the addition of 10% of Co- or Mn-based IL leads to a significant reduction of yield strain in the respective IGs. The latter IGs exhibit similar yield strain for all IL fractions. Table 4 summarizes stiffness and yield strains of all samples.

The temperature-dependent ionic DC-conductivity of the IGs was determined from broadband dielectric spectroscopy (BDS) measurements. The dielectric data are obtained from the complex dielectric permittivity

(1) ε*(ω) = ε’(ω) − iε’’(ω)

and the AC conductivity

(2) σ*(ω) = σ’(ω) + iσ’’(ω)

The DC conductivity σ_DC_ is obtained form the plateau region of the frequency-dependent real part of the conductivity. To that end, the minimum of the *d* log σ’/*d* log f at a specific temperature was used.

**Figure 8:**
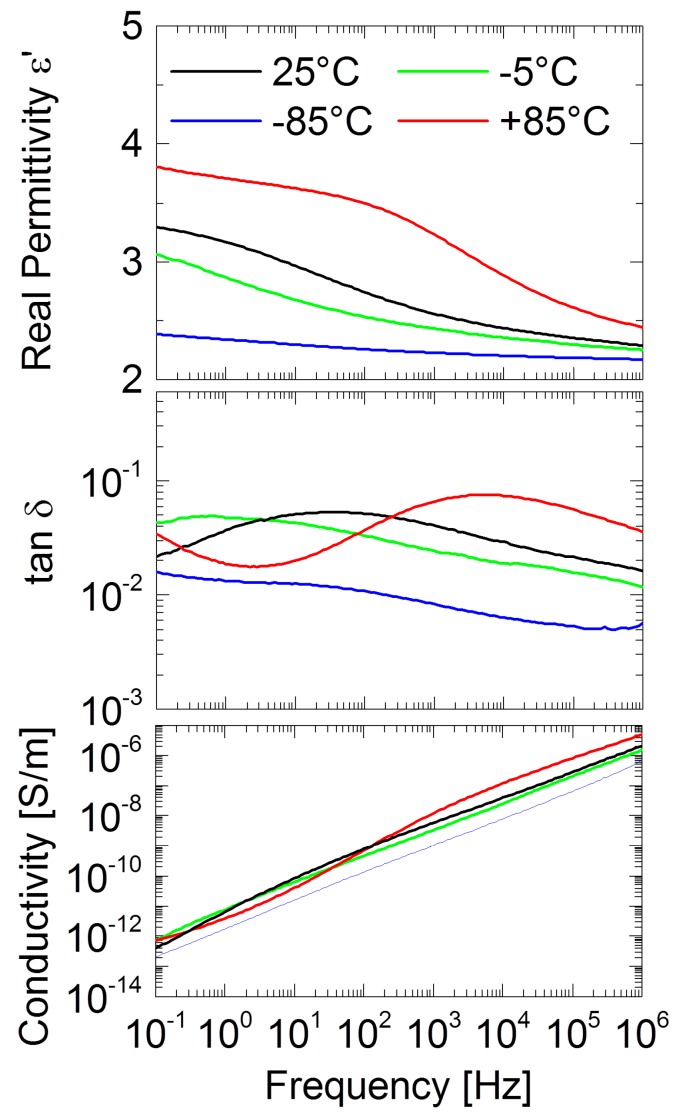
Relative permittivity, dielectric loss tangent and alternating current (AC) conductivity as function of temperature for pure PMMA.

Figure 8 reports the frequency-dependent permittivity, loss tangent (tan δ and real conductivity of PMMA *vs.* temperature. Drop cast PMMA shows a relative permittivity *e*_r_ of 2.55 at 1 kHz , which is comparable to literature [76]. A further increase of frequency shows a gradual decrease of the permittivity and the dielectric loss of tan δ resulting in values of *e*_r_ = 2.28 and tan δ = 0.02 at 1 MHz and 25 °C. This frequency-dependent decrease of the PMMA can be assigned to the ester group in PMMA (–COOCH_3_) which may cause orientation polarization (OP) in PMMA [76]. The divergence of relative permittivity and loss tan is associated with polymer chain segments not following the external field at higher frequencies [77]. The relative permittivity and loss tangent of pure PMMA show an increase with temperature to 3.4 and 0.065, respectively, for 1 kHz and 95 °C (Figure 8). This effect may be connected to OP due to increased movement of polymer chain segments at these higher temperatures [76], and possibly the presence of free charges.

**Figure 9:**
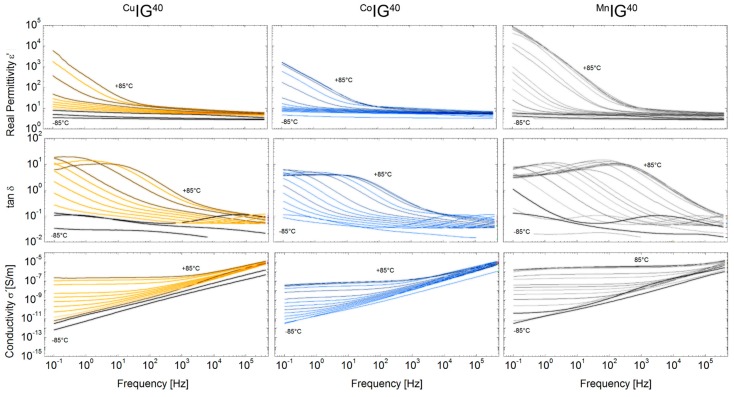
Relative permittivity and dielectric loss tangent as a function of temperature for the IGs with 40 vol % (^Cu^IG^40^, ^Co^IG^40^, ^Mn^IG^40^).

The increase of dielectric losses, especially at high temperatures and low frequencies, are attributed to orientation and interfacial relaxation processes (Figure 9). Tan d shows a shifting of the resident peak due to acting of the used ionic liquid. Compared to the measured glass transition via DSC (Table 4) the *T*_g_ of the IGs determined by dielectric measurements at 100 Hz are shifted to lower values.

## 3. Discussion

We have studied a set of new ionogels based on metal-containing ILs. The crystal structures of the ILs are identical to those already reported by Zhong *et al.* [67] and the ILs show classical IL behavior in that a melting transition is observed on first heating in the DSC, which is not visible on second and third heatings. This indicates that the ILs exhibit a rather strong undercooling, consistent with many other ILs [78]. The IGs were made using established protocols [38] yielding macroscopically homogeneous transparent and colored films. IR Spectroscopy (Figure 6) suggests that the interaction between the ILs and the PMMA is rather low and thus confirms data reported by other authors [38,79]. Interestingly, DSC shows two independent glass transitions in all IGs (Figure 4b). This indicates that the IL and the PMMA matrix do not mix well on a molecular scale. Indeed, optical microscopy shows colored IL droplets in an almost colorless matrix, the PMMA. This is in contrast to a previous example based on [Bmim][FeCl_4_], where DSC only shows one phase transition in the IGs thus suggesting that in this particular case, there is mixing on a molecular scale [38]. We currently speculate that either the differences in the metal ion charges (+2 in the current study *vs.* +3 in the previous case) or the overall charge of the IL anion (−2 in the current study *vs.* −1 in the previous case) could be responsible for differences in the IL-PMMA interactions. Alternatively, it may also be possible that the higher number of imidazolium cations in the current case (two per formula unit *vs.* one per formula unit in the previous case) may lead to changes in the mutual miscibility. Finally, as [Bmim][FeCl_4_] also has a very different crystal structure [80], it may also be possible that the crystallization of the ILs studied here is less affected by the presence of the surrounding polymer. Solid-state UV/Vis spectroscopy (Figure 5) detects no peak shift and the intensity of absorption increases with higher IL fraction in the IGs. The absorption maxima observed in the IGs show a hypsochromic shift of *ca.* 10 nm compared to the original IL. The solid-state UV/Vis spectra therefore suggest that either there is a confinement effect on the coordination geometry around the metal ion or there is a weak interaction between IL and PMMA centered around the metal ion. Alternatively, there could also be changes in the IL-IL interaction on confinement, similar to cation-induced geometry variations in [CuCl_4_]^2−^ and [CuBr_4_]^2−^ anions [81,82]. The apparent contradiction between IR (suggesting no interaction between IL and PMMA) and UV/Vis spectroscopy (possibly suggesting a weak interaction) is resolved because IR spectroscopy does not probe the M–Cl bonds whereas UV/Vis does: in essence these data therefore suggest that—if there is an interaction at all—it stems from the IL anion and the PMMA.

The mechanical data show a gradual decrease of Young’s modulus with increasing IL content and a corresponding decrease in the yield strain. Adapting the model of Lodge *et al.* [83] assuming that the polymer matrix acts like sponge filled by the ionic liquid (which is consistent with DSC, optical microscopy, and IR and UV/Vis spectroscopy, although on a micrometer length scale), the data presented here can be understood as follows. The increasing volume concentration of the ILs leads to a decreasing PMMA volume fraction. Hence, as the IL is present as individual droplets that do not contribute to the mechanical reinforcement of the material, the mechanical load is distributed on an ever-decreasing volume fraction of PMMA. As a result, IGs with higher IL fractions fail at lower loads and consequently exhibit lower Young’s moduli. This is consistent with the other data discussed above and with work from Li *et al.* [69], demonstrating that the energy dissipation in PMMA-based ionogels during fracture mainly arises from the fracture of carbon-carbon bonds. As there are less (PMMA) carbon-carbon bonds available to break at high IL loadings, these materials break earlier.

The same ionogel structure (IL droplets in a continuous matrix) is likely also responsible for the observed conductivity: in contrast to an earlier example [38] where the IL is homogeneously distributed in the IG (thus providing charge carrier transport throughout the entire IG) the current IGs exhibit a clear two-phase structure with IL-polymer phase boundaries that act as a barrier for the charge carriers. Therefore, the conductivity of the current IGs is lower by *ca.* two orders of magnitude when compared to IG based on [Bmim][FeCl_4_].

As a result, we propose that there is a series of morphology-controlled conductivity regimes in ionogels: (i) the highest conductivity can be found in well-ordered IL domains [84]; followed by (ii) disordered IL domains [84,85], which exhibit a lower conductivity; followed by (iii) the current system of isolated IL droplets in a continuous matrix; and finally (iv) the neat, non-conducting matrix, such as PMMA. These data furthermore suggest that IGs with the IL homogeneously distributed in the matrix, such as the example by Xie *et al.* [38], may have completely different mechanical properties due to the fact that these samples do not exhibit phase-separated IL and polymer domains, but this will need to be investigated in the future.

## 4. Materials and Methods

### 4.1. Materials

Acetonitrile (Sigma-Aldrich, Munich, Germany, ≥99%), Copper(II)chloride dihydrate (previously Fluka now Sigma-Aldrich, Munich, Germany, ≥99%), Cobalt(II)chloride hexahydrate (ucb, ≥99%), Manganese(II)chloride monohydrate, (Roth, Karlsruhe, Germany, ≥99%) , PMMA, (Sigma-Aldrich, Munich, Germany, average *M*_w_ ~120,000 by GPC) were used as received. Butyl-3-methyl-imidazolium chloride ([Bmim][Cl], Sigma-Aldrich, Munich, Germany, ≥95%) was recrystallized from the melt.

### 4.2. General Procedure for [Bmim]_2_[CuCl_4_], [Bmim]_2_[CoCl_4_] and [Bmim]_2_[MnCl_4_] Synthesis

The synthesis of [Bmim]_2_[CuCl_4_], [Bmim]_2_[CoCl_4_], and [Bmim]_2_MnCl_4_, was adapted from similar systems [63,64,65,86]. In a 250 mL round bottom flask 17.47 g (0.1 mol) of [Bmim][Cl] and 0.05 mol of the respective metal dichloride and 150 mL acetonitrile were mixed and stirred for 1 h at 75 °C. The acetonitrile was removed at 50 °C and 220 mbar. The resulting IL was dried for 24 h at 0.003 mbar.

### 4.3. IG Synthesis

In all cases 1 g of PMMA was dissolved in 10 mL of dry acetonitrile. To this solution 0.1, 0.2, 0.3, or 0.4 g of the respective ILs were added. The films were then made by slowly evaporation of the acetonitrile. For all prepared ionogels of PMMA with different IL the following abbreviations were used: ^Cu^IG^10^, ^Cu^IG^20^, ^Cu^IG^30^ and ^Cu^IG^40^ (10%–40% [Bmim]_2_[CuCl_4_]), ^Co^IG^10^, ^Co^IG^20^, ^Co^IG^30^ and ^Co^IG^40^ (10%–40% [Bmim]_2_[CoCl_4_]), ^Mn^IG^10^ , ^Mn^IG^20^, ^Mn^IG^30^ and ^Mn^IG^40^ (10%–40% [Bmim]_2_[MnCl_4_]).

### 4.4. Infrared Spectroscopy

ATR-IR spectra were obtained on a NEXUS FT-IR spectrometer (Thermo-Nicolet, Diamond, ATR correction was done via Omnic 8.1.11 (Thermo Fischer Scientific Germany BV & Co KG, Braunschweig, Germany).

### 4.5. UV/Vis Spectroscopy

Solid state UV/vis mearsurements were done on a PerkinElmer lambda 750 (PerkinElmer Life and Analytical Sciences, Shelton, CT, USA).

### 4.6. Magnetic Properties

Magnetic susceptibility was performed on a Magnetic Susceptibility Balance-Auto (MSB-Auto, Johnson Matthey GmbH, Redwitz, Germany).

### 4.7. Thermal Analysis

Thermogravimetric analysis (TGA) was done on a Netzsch TG209 F1 Libra (Netzsch-Gerätebau GmbH, Selb, Germany) with a heating rate of 10 K/min. DSC experiments were done on a Netzsch Polyma 214 (Netzsch-Gerätebau GmbH, Selb, Germany) under nitrogen atmosphere and a heating rate of 10 K/min The samples were weighed in aluminum pans with a pierced lid from the same manufacturer. Samples were measured over two heating and cooling cycles from −150 to 150 °C at 10 K·min^−1^. Isothermal time between heating and cooling cycles were 10 min.

### 4.8. Optical Microscopy

Optical micrographs were obtained on a Zeiss Primo Star (Carl Zeiss Microscopy GmbH, Jena, Germany) at ambient condition.

### 4.9. Dielectric Spectroscopy. IG Thickness

The thickness of the film was determined using a micrometer screw. It is found that all films had nearly identical thickness of approx. 125 µm.

### 4.10. Mechanical Characterization

A series of at least 5 mechanical tests were carried out at room temperature using a Zwick Z005 (Zwick GmbH & Co KG, Ulm, Germany) tensile testing machine equipped with a 2.5 kN load cell from the same manufacturer. The rectangular films were mounted to the tensile to allow uni-axial deformation. Steel clamps were used to fix the samples. The tests were performed at room temperature and a constant strain rate of 0.05%/s until the sample fractures. The Young’s modulus Y was determined from the slope in the linear region before the sample ruptures. The slopes of the initial portions of the stress-stretch curves are used to determine the moduli (Y). The Young’s modulus was determined from the nominal stress, the loading force divided by the initial cross-sectional of the un-deformed materials and the achieved deformations. The PMMA and the resulting composites are assumed to be incompressible such that the Poisson ratio is 0.5.

### 4.11. Dielectric and Electrical Characterization

Dielectric properties were determined using the impedance analyzer (Novocontrol Alpha frequency response analyzer) (Novocontrol Technologies GmbH & Co. KG, Montabaur, Germany) at a frequency range from 0.1 to 10 MHz at an applied voltage of 3 V. A Quatro Cryosystem from the same manufacturer was used to control the temperature within a cryostat with an accuracy better than of ±0.1 K. For dielectric measurements a temperature figure was applied form initial RT (25 °C) down to −85 °C followed by a stepwise increase with 10 K increments to 95°. The chosen temperature was held constant for 5 min for all studied samples before frequency scan. All IL films were equipped with circular frames of 20 mm diameter. The so-defined freestanding area was covered with gold powder and finally covered with the metal discs to ensure a proper electric contact to the measurement system.

## 5. Conclusions

In conclusion, we have successfully prepared ionogels by combining PMMA with different metal-containing ionic liquids. The IGs are transparent and mechanically robust. The novelty of this study lies in the fact that it has been possible to correlate the thermal, mechanical, and electrical behavior to the microstructure of the ionogel. The current ionogels are different from previous polymer-based examples in that here the IL is present as isolated IL droplets in a continuous matrix yielding a drastically different behavior. In summary, the data thus demonstrate that the microscale architecture (homogenous *vs.* phase-separated morphologies) is one key parameter that enables the adjustment of technologically relevant properties like Young’s modulus or conductivity in ionogels.

## Figures and Tables

**Table 1:** X-ray crystallographic and refinement data for [Bmim]_2_[CuCl_4_], [Bmim]_2_[CoCl_4_] and [Bmim]_2_[MnCl_4_].

Parameter	[Bmim]_2_[CuCl_4_]	[Bmim]_2_[CoCl_4_]	[Bmim]_2_[MnCl_4_]
Chemical Formula	C_16_H_30_Cl_4_CuN_4_	C_16_H_30_Cl_4_CoN_4_	C_16_H_30_Cl_4_MnN_4_
Formula weight	483.78	479.17	475.18
Temperature/K	150(2)	150(2)	150(2)
Crystal system	monoclinic	monoclinic	monoclinic
Space group	*Cc*	*Cc*	*Cc*
Unit cell dimension *a*/Å	14.1014(5)	14.3976(7)	14.4272(7)
Unit cell dimension *b*/Å	9.7074(4)	9.7151(6)	9.7344(3)
Unit cell dimension *c*/Å	17.1303(6)	16.8773(9)	16.9521(7)
β/°	107.431(3)	107.699(4)	107.563(3)
Volume/Å^3^	2237.25(15)	2249.0(2)	2269.78(16)
*Z*	4	4	4
Calculated density (ρ_calc_)/mg·m^−^³	1.436	1.415	1.391
μ/mm^−1^	1.461	1.246	1.060
*F*(000)	1004	996	988
Crystal size	1.0 × 1.2 × 1.4	0.05 × 0.2 × 0.4	0.2 × 0.3 × 0.4
Crystal color	orange	blue	colourless
Crystal description	block	plate	block
Theta range for data collection	2.49 to 24.99	2.53 to 25.00	2.52 to 25.00
Miller Index ranges	−16 ≤ *h* ≤ 16 −11 ≤ *k* ≤ 11 −20 ≤ *l* ≤ 20	−17 ≤ *h* ≤ 17 −11 ≤ *k* ≤ 11 −19 ≤ *l* ≤ 20	−17 ≤ *h* ≤ 17 −11 ≤ *k* ≤ 11 −20 ≤ *l* ≤ 19
Reflections collected	14160	9990	14352
Unique reflections	3937 (*R*_int_ = 0.0961)	3770 (*R*_int_ = 0.0279)	3866 (*R*_int_ = 0.0167)
Data/restraints/parameters	3937/2/227	3770/2/227	3866/2/227
Final *R* indices (*I* > 2σ(*I*))	*R*_1_ = 0.0316 *wR*_2_ = 0.0848	*R*_1_ = 0.0210 *wR*_2_ = 0.0517	*R*_1_ = 0.0155 *wR*_2_ = 0.0423
*R* indices (all data)	*R*_1_ = 0.0316 *wR*_2_ = 0.0848	*R*_1_ = 0.0231 *wR*_2_ = 0.0525	*R*_1_ = 0.0157 *wR*_2_ = 0.0423
Goodness-of-fit on F^2^	1.042	1.036	1.060
Largest diff. peak and hole/Å^3^	0.314/−0.431	0.253/−0.287	0.299/−0.329
CCDC	1452214	1452215	1452218

**Table 2:** Melting points, glass transitions, and magnetic moments of the three ILs and according to the literature (*).

IL	*T*_g_ (°C)	*T*_m_ (°C)	µ_eff_	(*) µ_eff_ [68]
[Bmim]_2_[CuCl_4_]	−48.6 ± 0.5	26.1 ± 0.5	1.78 ± 0.1	1.8–2.1
[Bmim]_2_[CoCl_4_]	−42.5 ± 1.4	61.5 ± 1.4	4.47 ± 0.1	4.3–5.2
[Bmim]_2_[MnCl_4_]	−49.2 ± 0.4	53.3 ± 0.4	5.42 ± 0.1	5.7–6.0

**Table 3:** *T*_g_ observed for PMMA, ^Cu^IG^10−40^, ^Co^IG^10−40^, and ^Mn^IG^10−40^.

IG	*T*g, onset/°C	*T*g, onset/°C
0 (Pure PMMA film)	-	108.0 ± 0.6
^Cu^IG^10^	−48.3 ± 0.3	116.6 ± 0.7
^Cu^IG^20^	−50.2 ± 0.8	114.3 ± 0.7
^Cu^IG^30^	−55.2 ± 0.6	114.7 ± 0.7
^Cu^IG^40^	−51.0 ± 0.7	115.9 ± 0.6
^Co^IG^10^	−47.4 ± 2.5	113.9 ± 2.4
^Co^IG^20^	−46.3 ± 1.6	114.4 ± 2.6
^Co^IG^30^	−44.9 ± 1.4	116.4 ± 2.3
^Co^IG^40^	−45.5 ± 1.3	114.8 ± 2.3
^Mn^IG^10^	−55.3 ± 2.4	114.0 ± 1.1
^Mn^IG^20^	−53.5 ± 0.4	114.5 ± 1.2
^Mn^IG^30^	−53.8 ± 0.7	113.6 ± 1.0
^Mn^IG^40^	−51.6 ± 0.6	114.7 ± 1.3

**Table 4:** *T*_g_ observed for PMMA, ^Cu^IG^10−40^, ^Co^IG^10−40^, and ^Mn^IG^10−40^.

ID	Young’s Modulus [GPa]	Yield Strain [%]
PMMA	1.8011 ± 120.9	2.3 ± 0.52
^Cu^IG^40^	0.8328 ± 211.8	1.00 ± 0.55
^Co^IG^40^	0.9344 ± 86.6	1.07 ± 0.19
^Mn^IG^40^	0.6956 ± 72.9	0.67 ± 0.24

## Supplementary Materials

Supplementary materials can be found at http://www.mdpi.com/1422-0067/17/3/391/s1.

## Abbreviations

The following abbreviations are used in this manuscript:

MDPI: Multidisciplinary Digital Publishing Institute
DOAJ: Directory of open access journals
TLA: Three letter acronym
LD: linear dichroism
