# Supplementary material for: Ionogels Based on Poly(methyl methacrylate) and Metal-Containing Ionic Liquids: Correlation between Structure and Mechanical and Electrical Properties
Source: Int J Mol Sci. 2016 Mar 16;17(3):391. doi: 10.3390/ijms17030391 (PMC4813247; doi:10.3390/ijms17030391)
Supplement: Supplementary file 1 [file ijms-17-00391-s001.zip › ijms-121599-supplementary-publish.docx]

Supplementary Materials: Ionogels Based on Poly(methyl methacrylate) and Metal-Containing Ionic Liquids: Correlation between Structure and Mechanical and Electrical Properties

Kerstin Zehbe, Matthias Kollosche, Sebastian Lardong, Alexandra Kelling, Uwe Schilde and Andreas Taubert

Single crystal X-ray structure determinations of compounds 1–3.

**General information**

Suitable single crystals of were mounted on a glass fibre for data collection on an Imaging Plate Diffraction System IPDS-2 (STOE) at 110 K with graphite-monochromated Mo-K_α_ radiation (*λ* = 0.71073 Å). The data were corrected for Lorentz, polarization and extinction effects. The structures were solved with SHELXS-2013/1^[[1]](#footnote-1)^ using direct methods and refined against *F*^2^ by means of full-matrix least-squares procedures with SHELXL-2013/2.^[[2]](#footnote-2)^ The non-hydrogen atoms were refined anisotropically. All hydrogen atoms were calculated in their expected positions and refined using a riding model with C-H = 0.98 Å (CH3), 0.99 Å (CH2), 0.95 Å (Carom), and with U_iso_(H) = 1.2Ueq(C) with the exception of methyl hydrogen atoms, which were refined with U_iso_(H) =1.5Ueq(C). For the visualization of the structures the graphic programs DIAMOND^[[3]](#footnote-3)^ and ORTEP-3^4^ were used. CCDC-123456 (1), CCDC-123457 (2) CCDC-123458 (3) contain the supplementary crystallographic data for this paper. These data can be obtained free of charge from the Cambridge Crystallographic Data Centre via www.ccdc.cam.ac.uk/data_request/cif.


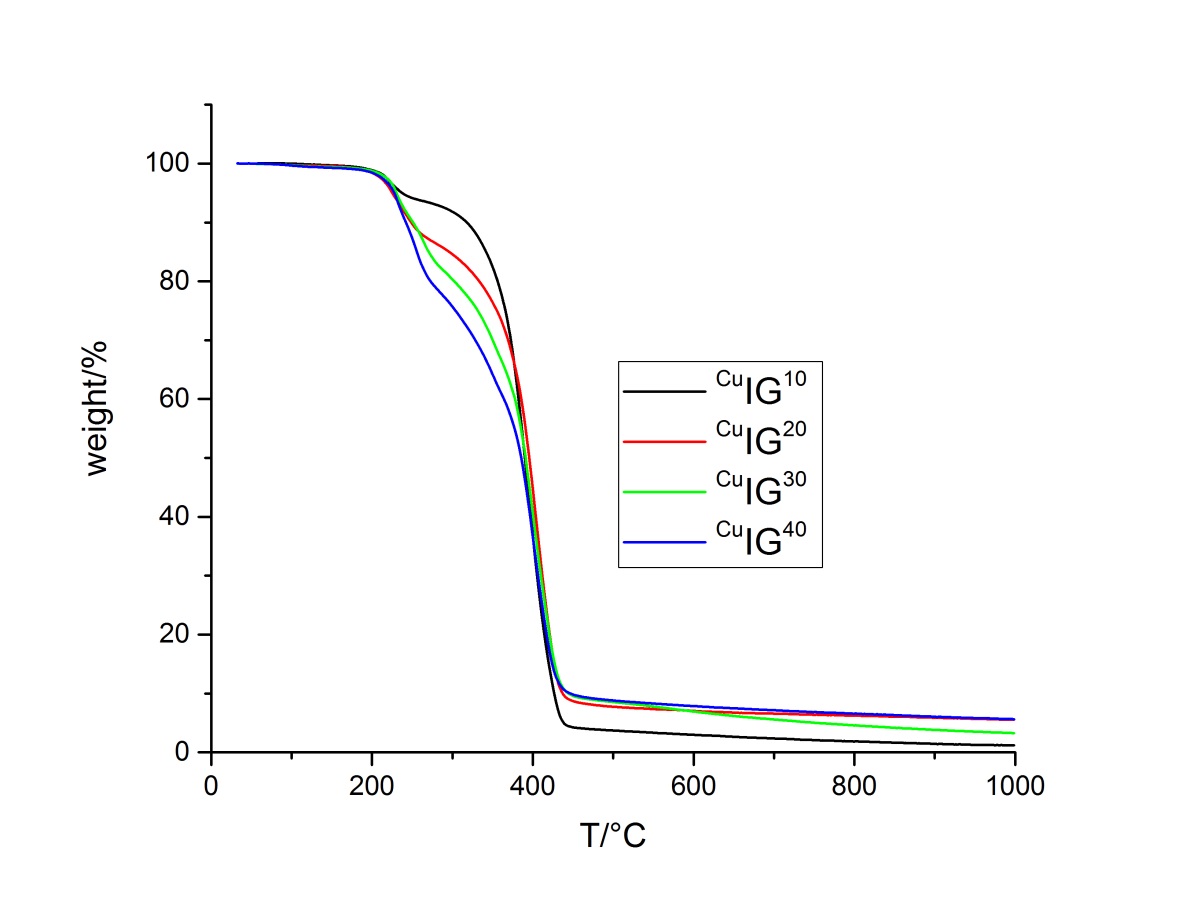


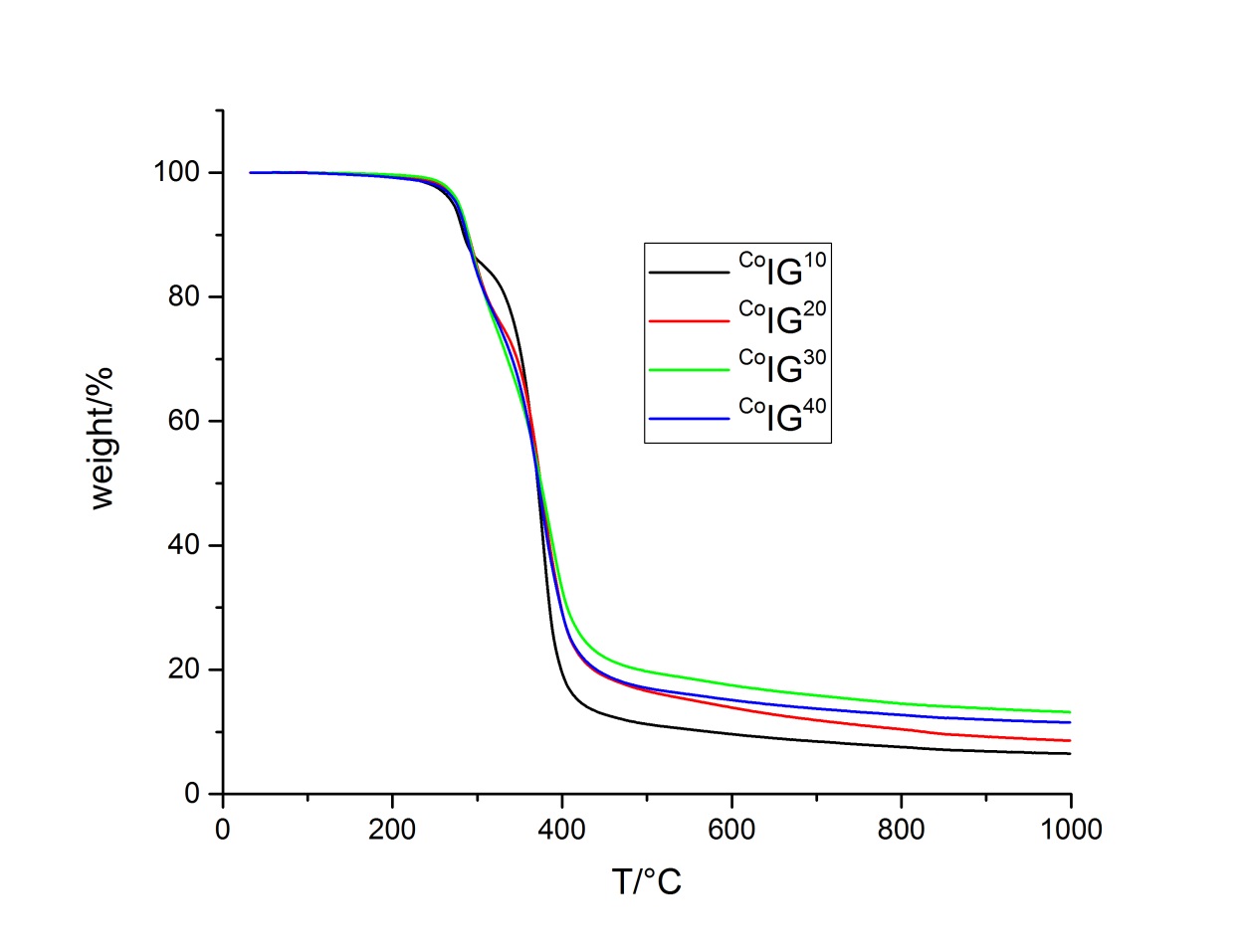


**Figure S1.** TGA-curves of ionogels with different fractions of [Bmim]_2_[CuCl_4_] (^Cu^IG^10–40^) and [Bmim]_2_[CoCl_4_] (^Co^IG^10–40^) Measurements under nitrogen, heating rate is 10 K/min.

1. Sheldrick, G. M. SHELXS-2013/1, Program for the Crystal Structure Solution, Universität Göttingen, Göttingen (Germany), 2013. [↑](#footnote-ref-1)
2. Sheldrick, G. M. SHELXL-2013/2, Program for the Crystal Structure Refinement, Universität Göttingen, Göttingen (Germany), 2013. [↑](#footnote-ref-2)
3. Diamond, Vers. 4.0.1, Crystal Impact, 2014.

   ^4^ Farrugia, L. J. ORTEP-3 for WINDOWS - version of ORTEP-III with a graphical user interface, J. appl. Cryst. 30 (1997) 565. [↑](#footnote-ref-3)
